# Evaluation of Growth Patterns and Body Composition in C57Bl/6J Mice Using Dual Energy X-Ray Absorptiometry

**DOI:** 10.1155/2014/253067

**Published:** 2014-07-10

**Authors:** Sara Gargiulo, Matteo Gramanzini, Rosario Megna, Adelaide Greco, Sandra Albanese, Claudio Manfredi, Arturo Brunetti

**Affiliations:** ^1^Institute of Biostructures and Bioimages of National Council of Research of Naples, Via De Amicis 95, 80145 Naples, Italy; ^2^CEINGE-Biotecnologie Avanzate Scarl, Via G. Salvatore 486, 80145 Naples, Italy; ^3^Department of Advanced Biomedical Sciences, Federico II University, Via Pansini 5, 80131 Naples, Italy; ^4^Department of Neurosciences, Reproductive and Odontostomatological Sciences, Federico II University, Via Pansini 5, 80131 Naples, Italy

## Abstract

The normal growth pattern of female C57BL/6J mice, from 5 to 30 weeks of age, has been investigated in a longitudinal study. Weight, body surface area (BS), and body mass index (BMI) were evaluated in forty mice. Lean mass and fat mass, bone mineral content (BMC), and bone mineral density (BMD) were monitored by dual energy X-ray absorptiometry (DEXA). Weight and BS increased linearly (16.15 ± 0.64–27.64 ± 1.42 g; 51.13 ± 0.74–79.57 ± 2.15 cm^2^, *P* < 0.01), more markedly from 5 to 9 weeks of age (*P* < 0.001). BMD showed a peak at 17 weeks (0.0548 ± 0.0011 g/cm^2^∗ m, *P* < 0.01). Lean mass showed an evident gain at 9 (15.8 ± 0.8 g, *P* < 0.001) and 25 weeks (20.5 ± 0.3 g, *P* < 0.01), like fat mass from 13 to 17 weeks (2.0 ± 0.4–3.6 ± 0.7 g, *P* < 0.01). BMI and lean mass index (LMI) reached the highest value at 21 weeks (3.57 ± 0.02–0.284 ± 0.010 g/cm^2^, resp.), like fat mass index (FMI) at 17 weeks (0.057 ± 0.009 g/cm^2^) (*P* < 0.01). BMI, weight, and BS showed a moderate positive correlation (0.45–0.85) with lean mass from 5 to 21 weeks. Mixed linear models provided a good prediction for lean mass, fat mass, and BMD. This study may represent a baseline reference for a future comparison of wild-type C57BL/6J mice with models of altered growth.

## 1. Introduction

Growth curve models are routinely used in biomedical research to better understand the overall development of body components and for studying disorders of growth and mechanisms involved in metabolic regulation. The laboratory mouse (Mus musculus) is an excellent tool to define the physiological parameters related to growth processes and to study the effects of genetic and nongenetic factors on a variety of metabolic events. Wild type or genetically engineered C57BL/6J mice are the most widely used inbred strain in several research fields, including cardiovascular diseases and atherosclerosis, developmental and skeletal biology, or endocrine diseases like diabetes [1–5]. In particular, female gender is employed to study major public health disorders of body composition like osteoporosis and obesity [6, 7]. For proper comparisons, a comprehensive survey of the normal pattern of overall body growth in this strain is needed. Indeed, changes in body composition related to age of wild type animals provide a reference standard to evaluate the effects of genetic manipulation, dietary factors, or pharmacological treatments. Moreover, the accurate phenotyping of translational preclinical systems with innovative technologies and noninvasive approaches is nowadays desirable. Several surveys of body weight and composition on a mouse strain have been conducted [8–14]. However, these studies have not provided comprehensive information about overall body composition. They are focused on models of diseases, have not used longitudinal data, and have generally been limited to a subset of age. DEXA currently provides a precise approach to assess body composition in murine models in a noninvasive and serial way, in the research fields of nutrition, metabolism, and bone physiology [15, 16]. DEXA has the advantages of low cost, low radiation, and short scan times, making it a practical method for assessing body composition in longitudinal studies. The objective of this retrospective study was to investigate general aspects of growth in healthy female C57BL/6J mice fed and housed under controlled laboratory conditions from peripuberal period to adult age. Total BMC and BMD, lean mass, and fat mass were measured by DEXA. Our results may serve as reference dataset for normal body weight and composition in the C57BL/6J mouse strain and assist in the comparison of wild-type animals with models of growth or altered body composition for future studies.

## 2. Materials and Methods

We performed a retrospective study involving females C57BL/6J examined by DEXA as control subjects. All experimental procedures have complied with the Italian D.L. n°116 of 1992 and associated guidelines in the European Communities Council Directive 2010/63/EU.

### 2.1. Mice and Housing Conditions

Forty female C57BL/6J mice were purchased from Harlan or Charles River Laboratories and shipped to our institution 1 week before the start of experimental procedures. All mice were housed in plexiglas cages sized 365 × 207 × 140 mm, surface 530 cm^2^, each cage containing 5 animals, according to standard laboratory conditions with an ambient temperature of 24°C and 14 h of light per day. Mice had free access to water and standard chow (Mucedola srl, Italy). Chemical analysis of the diet was 20% protein and 9% fat. The general health and well-being of the mice were monitored by veterinary staff with careful observation of activity, nest building, and interaction with cage mates and by physical examination, providing an assessment of the animals' hydration, body condition, and observable abnormalities. The morphometric variables and body composition were recorded. The number of observations for each age-group is provided in Table 1. Ten mice were examined only at 13 weeks of age, whereas longitudinal monitoring by DEXA was performed for twenty mice from 5 to 9 weeks of age (group 1), for five mice from 5 to 21 weeks of age (group 2) and for five mice from 13 to 30 weeks of age (group 3).

### 2.2. Growth Parameters Recording

Individual weights were taken on all mice at 5, 9, 13, 17, 21, 25, and 30 weeks of age to the nearest 0.1 gram using an electronic balance. Body length was determined by measuring nasal-to-anal distance to the nearest 0.1 mm using a caliper when mice were anesthetized and immediately after DEXA scan, in order to avoid inaccuracy from movement and to confirm with X-ray images the precise positioning of mice.

### 2.3. DEXA

**Figure 1:**
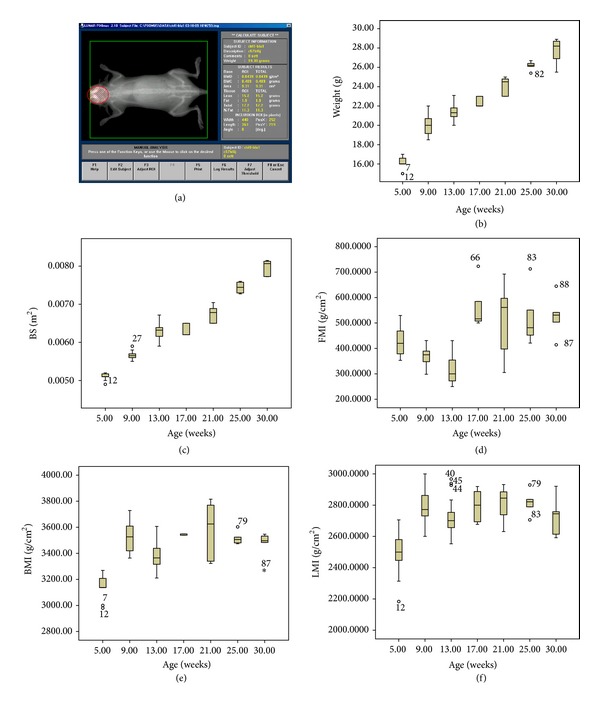
Total body DEXA assessment of body composition (a), changes of weight (b), surface area (c), fat mass index (d), body mass index (e), and lean mass index (f) from 5 to 30 weeks of age in female C57BL/6J mice.

Changes in body composition were determined by DEXA using a dedicated densitometer (Lunar Piximus, GE Medical Systems Madison, WI). This system employs a cone beam X-ray source generating energies of 35 and 80 keV and a flat 100 × 80 mm detector having individual pixel dimensions of 0.18 × 0.18 mm. The ratio of energy attenuation in the luminescent panel separates bone, lean tissue, and fat mass. A quality control procedure was routinely performed with a calibration phantom before imaging. Animal care and anesthesia were conducted according the guidelines that we have described elsewhere [17]. Mice were anesthetized with intraperitoneal (IP) injection of 40 mg/kg ketamine and 0.8 mg/kg medetomidine, to ensure good immobilization and positioning during a five-minute acquisition [18]. At the end of the exam, mice were awakened with 1 mg/kg atipamezole IP. The following data were provided by the scanner: weight of lean tissue (Lean, g), weight of fat tissue (Fat, g), bone mineral density (BMD, g/cm^2^), and bone mineral content (BMC, g) (Figure 1(a)).

### 2.4. Data Analysis

Body surface area was derived from the DuBois equation:

(1) body  surface(m2)=0.007184×weight(kg0.425) ×height(cm0.725).

BMI was calculated as the ratio between body weight and square surface area (g/m^2^).

The growth rate,defined as the weight increment per unit of time, was derived from the formula:

(2) $$\begin{array}{c} R_{G}=\frac{\left(W2-W1\right)}{\left(t2-t1\right)} \end{array}$$

in which (*W*2 − *W*1) is the weight increment in the time interval (*t*2 − *t*1).

From the body composition parameters measured with the proprietary analysis software (Figure 1(a)), the following derivative values were calculated:

- the correct projected areal bone mineral content (BMC corr, g/m^2^): BMC (g)/BS;
- the correct projected areal bone mineral density (BMD corr, g/cm^2^∗m): BMD (g/cm^2^)/$\sqrt{\text{BS}}$;
- lean mass index (LMI, g/cm^2^): Lean (g)/BS (cm^2^);
- fat mass index (FMI, g/cm^2^): Fat (g)/BS (cm^2^).

Descriptive data were presented as mean ± SD and summarized in tables or plotted in graphs. Nonparametric Friedman's ANOVA was performed to compare longitudinal measurements collected at 13–21–25–30 and at 9–13–17–21 weeks, whereas the Wilcoxon-signed rank test was used for the serial data at 5 and 9 weeks. Spearman's rho or Kendall's tau nonparametric correlations were used to explore the relationships between DEXA values (lean mass and fat mass) and somatometric measurements, depending on the group size and in case of nonnormal distribution. A general linear mixed method, with 95% confidence intervals (CI) around the slopes and intercepts, was used to model the effects of predictor variables on changes in BMD, lean mass, and fat mass. These models incorporated a random intercept term to account for the correlation among repeated measures. The use of mixed models with in vivo studies is valid in improving the quality of the data analysis with small samples. In this way, mixed models also help to reduce the number of animals used in research [19, 20]. Statistical analysis was carried out using the software SPSS 18.0.2. (SPSS, Chicago, IL). All statistical tests were two-sided and values less than 0.05 were considered statistically significant.

## 3. Results

**Figure 2:**
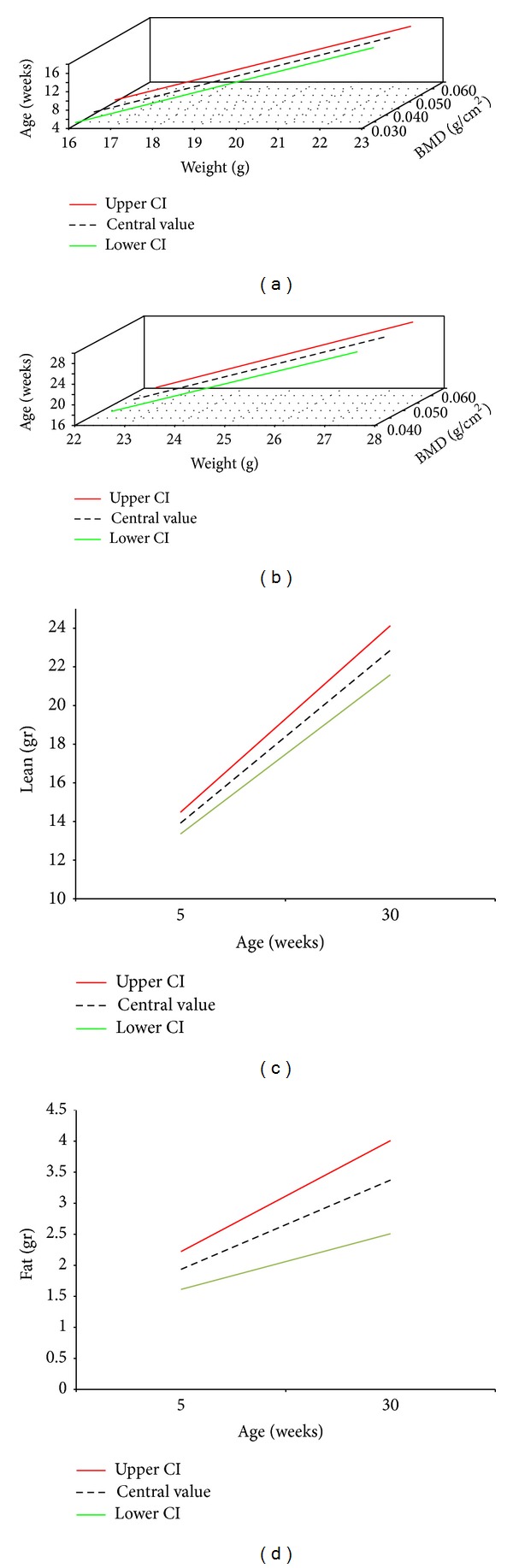
Graphical representation of the modelling results (predicted values with the 95% upper and lower confidence intervals): BMD from 5 to 17 weeks (a), BMD from 17 to 30 weeks (b), lean mass (c), and fat mass from 5 to 30 weeks in female C57BL/6J mice.

Descriptive and longitudinal statistics are given in Table 1. Weight and body surface area showed a linear increase from 5 to 30 weeks of age (Figures 1(b)-1(c)). Our data are in agreement with the typical correlation between body weight and age for mice maintained in production colonies at The Jackson Laboratories available from the Mouse Phenome Database (MPD, http://www.jax.org/phenome). The absolute growth curve of weight against time appears U-shaped and is characterized by two phases of major increment: from 5 to 9 weeks of age (1 g/week) and from 21 to 25 weeks of age (0.69 g/week); from 9 weeks of age the gain of weight per unit of time decreases; it occurs at the age of puberty, but the mature weight, defined as the weight after which no further rise occurs, is not yet reached at 30 weeks of age. BMC described a sigmoid curve, with an initial phase of rapid increase from 0.285 ± 0.075 g (5 weeks) to 0.394 ± 0.025 g (9 weeks of age) (*P* < 0.001), an intermediate exponential phase from 9 to 21 weeks (*P* < 0.01), and a subsequent stationary phase. Correct projected areal bone mineral content (BMC corr, g/cm^2^) had a bell-shaped curve, with a peak and inflection point at 17 weeks of age (73.65 ± 4.88, *P* < 0.01). The peak BMD was reached at 17 weeks of age (0.0548 ± 0.0011 g/cm^2^∗ m), with an increase from 5 to 17 weeks of age, after which it showed a slow decline until 30 weeks of age. Correct projected areal bone mineral density (BMD corr, g/cm^2^∗ m) showed a peak at 17 weeks of age (0.69 ± 0.02, *P* < 0.01). Lean mass and fat mass increased progressively, with an evident gain in body protein at 9 weeks (15.8 ± 0.8 g/cm^2^) and 25 weeks (20.5 ± 0.3 g/cm^2^) of age, and a prevalence of fat mass increases from 13 (2.0 ± 0.4 g/cm^2^) to 17 (3.6 ± 0.7 g/cm^2^) weeks of age (16.92% of body weight). LMI described a flattened linear pattern that reached the maximum value at 21 weeks of age (0.284 ± 0.009 g/cm^2^) (Figure 1(c)). BMI increased from 3.16 ± 0.08 g/cm^2^ (5 weeks) to 3.52 ± 0.11 g/cm^2^ (9 weeks) (*P* < 0.001), peaking at 21 weeks (3.57 ± 0.02, *P* < 0.01), followed by a plateau from 25 to 30 weeks (Figure 1(e)). FMI showed a bell-shaped curve which peaks at 17 weeks (0.058 ± 0.009 g/cm^2^) (Figure 1(f)). Lean mass showed a moderate positive correlation with BMI, weight, and body surface area from 5 to 21 weeks of age, varying between 0.45 and 0.85, with the strongest relationship at 9 weeks and the weakest one at 17 weeks of age, whereas bivariate analysis did not show any significant correlation between fat mass and somatometric indices (Table 2). Since in our sample the pattern of change in BMD with age does not show a unique slope, but it reaches a peak at about 17 weeks of age and then declines, we fitted a linear model from the prepuberal age to peak BMD and another one from peak BMD until to 30 weeks of age. The final regression equations for the prediction of changes in BMD, lean mass, and fat mass are summarized in Table 3 and shown in Figure 2. The longitudinal data represented in Table 4 include the means and standard deviations of BMD, lean mass, and fat mass in three experimental groups repeatedly scanned by DEXA at different age ranges.

## 4. Discussion

The introduction of murine models in biomedical research has increased the need for noninvasive methods to study their phenotype. The evaluation of changes in body composition and weight with age of wild type mice gives a standard with which to compare the effects of genetic manipulation, pharmacological, and surgical treatments or nutrition [9]. For example, the knowledge of a normal growth pattern provides a reference to study the etiology of growth retardation in mouse models of cystic fibrosis [21] or models of enhanced growth [9]. Investigations about the normal murine growth curve can be also useful to understand the molecular basis of therapeutic agents such as somatotropin [22] or human growth hormone gene therapy [23] and their effects on body composition. The C57BL/6J strain has increasingly been used as a tool to study bone metabolism as well as estrogen action on bone and for the development of pharmaceutical, nutritional, or mechanical interventions for bone remodeling and osteoporosis. The availability of different genetically modified murine models with potentially interesting bone phenotypes increased also the demand for noninvasive methods to effectively compare their differences during growth and development. In addition to the study of skeletal growth and regulation [12, 24, 25], C57BL/6J mice have been widely used as a background strain in other research fields such as the study of adipose tissue metabolism [11, 26], diet-induced obesity [27], and endocrine diseases influencing the metabolism of lipid, carbohydrate, and protein such as metabolic syndrome, type 2 diabetes [9, 28], or Prader-Willi syndrome [29]. It has generally been suggested to use only one sex in these experiments to avoid complications arising from this source because there are differences both in magnitude and in change of body components between young males and females [30–33]. Total animal growth or organ growth had been measured in terms of weight dimension, deoxyribonucleic acid (DNA), ribonucleic acid (RNA) and protein contents of whole body or single organs, NMR metabolomics, serum hormones dosages, and tissue composition [21, 26, 29, 31] measured by invasive and/or* ex vivo* destructive methods. Similarly, changes in bone parameters can be evaluated in mice by invasive and/or time consuming methods including histomorphometry, biochemistry of bone tissue, and biochemical markers of skeletal turnover in blood and urine. The chemical carcass analysis is considered the “gold standard” for determining body composition [11, 29] and high resolution micro-CT and histomorphometry of bone samples are the reference techniques to measure skeletal biomechanical properties [8, 25, 34]. Nevertheless, DEXA is considered a versatile technique for the evaluation of body composition, because it is simple, fast, noninvasive, and accurate. This technique is more readily accessible and less expensive than X-ray computed tomography (CT) and nuclear magnetic resonance (NMR) and can measure bone and soft tissues composition in the whole body and in specific regions [16]. Dedicated small animal densitometers currently provide a precise approach to assess body composition in murine models in a noninvasive and serial way [15]. Mineralized tissues are classified as bone, while nonskeletal tissue is assigned to fat and lean compartments on the base of the differential attenuation of low and high energy X-rays. In a review of literature, values of fat and lean tissue obtained by DEXA were compared favorably to those values obtained by NMR and CT scan [16, 35–37]. The precision of a particular DEXA device for assessing whole body composition is generally good with coefficients of variation of about 1% for BMC and 2-3% for total body fat [38]. Like other imaging techniques, it can also contribute to improve the well-being of animals and to reduce the number of laboratory animals used in accordance with the principles of “refinement” and “reduction” declared by Russell and Burch in 1954. An issue among body composition specialists is how interindividual comparison of body composition parameters should be made: absolute value (weight measurement units)* versus* relative value (i.e, percentage of body weight) or normalized value for body size (i.e, typically height squared or body surface area). Therefore, absolute values of bone mineral content, lean and fat mass can be used to evaluate nutritional status by comparing individuals with themselves or with reference values, whereas the use of normalized indices has the advantage of compensating for interindividual differences in body size. From a methodological viewpoint, it is generally assumed in medicine that correction for interindividual body size differences is needed to avoid mistakes in the case of dimensional-dependent variables. It has been demonstrated that BMD in g/cm^2^ depends on bone size [39] and in clinical practice it is assumed that body lean and fat should be correlated with body size variables such as weight or height [40]. The formula for body surface area includes both weight and body length and it is reported that this variable of body size shows the highest correlation with BMD. For these reasons, the whole body measures in our study were also normalized by this parameter, providing simple indexes to classify the normal body composition in C57BL/6J female mice. Moreover, the International Society for Clinical Densitometry (ISCD) recommends the use of total body's parameters less head in growing subjects. As a result, we developed subtotal measures of BMD, BMC, lean mass, and fat mass (whole body less head) to create reference curves in subjects between 5 and 30 weeks of age, normalized to body size (body surface area). Finally, the availability of repeated measures could provide a more accurate evaluation of within-subject variability over time and an increment of statistical power, reducing the cost, the time, and the animals used in a study. Nevertheless, in spite of the advantages over cross-sectional designs, longitudinally collected data require special techniques for analysis because they often have unbalanced data and because repeated measurements taken on the same individual are correlated with each other. We investigated the functional relationship between BMD, lean mass, fat mass, and age to construct a normal reference database for C57BL/6J strain. Bone mineral density is the most important measurable determinant of bone health status and its age-related changes are a significant issue, especially in women, to evaluate skeletal diseases. Defining thresholds in comparison to a young adult population has proven itself a useful tool in the field of osteoporosis research (e.g, the bone mineral density T-score). At the moment, the general pattern of age-related changes of BMD in normal C57BL/6J female mice has not been clearly delineated. In this study, the BMD by age curve was linear from 5 to 17 weeks of age with a progressive BMD increase followed by a slight decline of BMD between 21 and 30 weeks. BMI is a mathematical relationship of a subject's weight with height or other indices of body size like the body surface area. BMI is used as a screening tool for overweight and obesity, and high BMI-for-age values are related to clinical risk factors for chronic diseases including hyperlipidemia, elevated insulin, and high blood pressure. BMI has been shown to be an approximate indicator of lean mass and fat mass and some clinical research has shown that it correlates with dual energy X-ray absorptiometry measures [41, 42]. Nevertheless, BMI does not consider the composition of body tissues and it can be misleading if subjects have a relatively high content of lean tissue. Moreover, owing to inherent differences in body shape between mice and humans, using BMI to evaluate obesity in mice models can be tricky. In humans, BMI is calculated from weight in kilograms divided by height in square meters (kg/m^2^) and it has been described in rodent models of obesity using body weight and nose-to-anus length body (g/cm^2^) [43–46]. To our knowledge, correlation between somatometric measures and DEXA parameters in mice has not been reported comprehensively in literature and there are no clear thresholds for BMI that define healthy, overweight, and obese states in this species. In our experimental sample, the relationship between BMI and lean mass was found to be moderate, whereas the association between BMI and body fat was weak. Using LMI and FMI, abnormalities in lean or fat mass can be assessed without interference from other body components and one can judge whether the deficit or excess of body weight is selectively due to a change in lean mass versus fat mass or both combined. A normal database may be useful for the evaluation of a deficit in fat-free mass with or without excess fat mass (sarcopenic obesity) for a given age category, complementing the classical concept of body mass index in a more qualitative manner. Up to now, reference ranges for LMI and FMI in healthy C57BL/6J female mice as a function of age have not been clearly defined. The comparison of a subject's FMI value to FMI values of healthy ones may be useful in the diagnosis and management of obesity models. In conclusion, our data describe body composition in C57BL/6J mice, adding useful reference values for this strain in different research settings. Limitations of the present study may include a relatively small sample size from 17 to 30 weeks of age, even if repeated measures are available. Moreover, a likely contributor to the variance was the differences among size of litters and we did not control for this variable. On the other hand, size of litters is partially determined by genetic effects, thus random balance of this variable among the groups may be useful to dilute heritability. Nevertheless, the data presented here may provide a comprehensive reference standard for body growth and composition on a specific murine strain using well established DEXA technology. These results could be helpful in biomedical research to evaluate a variety of abnormalities of bone mineralization and metabolic diseases in mice models.

## Figures and Tables

**Table 1:** Mean and standard deviation values for morphometric and body composition measurements in female C57BL/6J mice from 5 to 30 weeks of age.

Age (weeks)	5	9	13	17	21	25	30
Female number	20	20	20	5	10	5	5

Body weight, g	16.15 ± 0.64	19.98 ± 0.91**	21.33 ± 0.67	22.40 ± 0.54	24.04 ± 0.90*	26.16 ± 0.48	27.64 ± 1.42*
Body surface area, cm^2^	51.13 ± 0.74	56.66 ± 0.94**	63.00 ± 1.98	63.20 ± 1.64	67.30 ± 0.89*	74.38 ± 1.52	79.57 ± 2.15*
BMI, g/cm^2^	3.16 ± 0.08	3.52 ± 0.11**	3.38 ± 0.10	3.54 ± 0.01	3.57 ± 0.02*	3.51 ± 0.05	3.47 ± 0.01
BMC, g	0.285 ± 0.075	0.394 ± 0.025**	0.438 ± 0.024	0.465 ± 0.025	0.447 ± 0.035*	0.467 ± 0.028	0.468 ± 0.054
BMC corr, g/m^2^	55.85 ± 15.27	69.62 ± 4.93	69.60 ± 11.38	73.65 ± 4.88	68.50 ± 3.43*	62.88 ± 4.55	58.96 ± 7.13
BMD, g/cm^2^	0.0391 ± 0.0042	0.0460 ± 0.0019**	0.0483 ± 0.0015	0.0548 ± 0.0011	0.0511 ± 0.0023*	0.0528 ± 0.0011	0.0510 ± 0.0049
ΔBMD/1 week age, %		+4.4	+1.3	+3.4	−1.7	+0.80	−0.7
BMD corr, g/cm^2^∗m	0.55 ± 0.06	0.61 ± 0.02	0.60 ± 0.02	0.69 ± 0.02	0.62 ± 0.03*	0.61 ± 0.18	0.57 ± 0.05
LM, g	12.8 ± 0.7	15.8 ± 0.8**	17.2 ± 0.8	17.7 ± 0.6	17.8 ± 1.2	20.5 ± 0.3	21.7 ± 1.4*
ΔLM/1 week age, %		+5.9	+2.2	+0.7	+0.1	+3.8	+1.2
LMI, g/cm^2^	0.250 ± 0.012	0.279 ± 0.010**	0.264 ± 0.005	0.279 ± 0.010	0.284 ± 0.009	0.281 ± 0.008	0.272 ± 0.013*
FM, g	2.2 ± 0.3	2.1 ± 0.2	2.0 ± 0.4	3.6 ± 0.7	3.7 ± 0.7*	3.9 ± 0.9	4.2 ± 0.7*
FM, %	14.90 ± 1.70	11.74 ± 1.00**	10.45 ± 1.74	16.92 ± 2.31	15.70 ± 3.53*	15.70 ± 3.17	16.19 ± 2.37
ΔFM/1 week age, %		−1.1	−1.2	+20.0	−0.7	1.4	+1.5
FMI, g/cm^2^	0.043 ± 0.005	0.037 ± 0.003**	0.040 ± 0.005	0.058 ± 0.009	0.045 ± 0.012*	0.052 ± 0.011	0.052 ± 0.008

*P ≤ 0.01; **P < 0.001; Wilcoxon-signed rank test (5–9 weeks) and nonparametric Friedmans ANOVA (9–13–17–21/13–21–25–30 weeks) on serial data.

BMI: body mass index; BMC: bone mineral content; BMC corr: correct projected areal bone mineral content; BMD: bone mineral density; BMD corr: correct projected areal bone mineral density; LM: lean mass; LMI: lean mass index; FM: fat mass; FMI: fat mass index, Δ : mean value difference/1-week age.

**Table 2:** Bivariate correlation coefficients Spearman's rho or Kendall's tau and *P* values (in round brackets) among DEXA soft tissues measurements and somatometric indexes in female C57BL/6J mice from 5 to 30 weeks of age.

5 weeks, no. 20	LM	FM
BMI	0.552 (0.014)	0.307 (0.201)
Body weight	0.552 (0.014)	0.307 (0.201)
Body surface area	0552 (0.014)	0.307 (0.201)

9 weeks, no. 20	LM	FM

BMI	0.661 (0.002)	0.239 (0.320)
Body weight	0.737 (0.0003)	0.145 (0.541)
Body surface area	0.761 (0.0001)	0.067 (0.780)

13 weeks, no. 25	LM	FM

BMI	0.451 (0.020)	0.531 (0.006)
Body weight	0.857 (<0.000001)	0.170 (0.410)
Body surface area	0.357 (0.080)	−0.417 (0.038)

17 weeks, no. 5^§^	LM	FM

BMI	0.499 (0.004)	0.369 (0.030)
Body weight	0.133 (0.460)	0.089 (0.632)
Body surface area	0.258 (0.772)	−0.335 (0.057)

21 weeks, no. 10	LM	FM

BMI	0.813 (0.004)	−0.605 (0.062)
Body weight	0.699 (0.024)	−0.280 (0.431)
Body surface area	0.873 (0.0009)	−0.711 (0.021)

25 weeks, no. 5^§^	LM	FM

BMI	0.000 (1.000)	0.599 (0.220)
Body weight	0.000 (1.000)	0.200 (0.806)
Body surface area	−0.223 (0.794)	0.200 (0.806)

30 weeks, no. 5^§^	LM	FM

BMI	0.800 (0.086)	0.316 (0.613)
Body weight	0.800 (0.086)	0.316 (0.613)
Body surface area	0.516 (0.386)	0.680 (0.236)

LEGEND:

^§^Kendall's tau test.

BMI: body mass index.

LM: lean mass.

FM: fat mass.

**Table 3:** Mixed model regression equations expressing body composition parameters in female C57BL/6J mice from 5 to 30 weeks of age.

Parameter	Equation	95% CI			*P* value	
		Intercept	Age	Weight	Age	Weight
BMD, g/cm^2^						
(5–17 weeks)	0.02258 + (0.00081 × age)+(0.00079 × weight)	0.01444 0.03072	0.00052 0.00100	0.00027 0.00131	0.0001	0.004
(17–30 weeks)	0.04220 − (0.00064 × age)+(0.00101 × weight)	0.03192 0.05247	0.00109 0.00019	0.00032 0.00170	0.006	0.004

LM, g (5–30 weeks)	12.128 + (0.358 × age)	11.706 12.550	0.32932 0.38591		0.0001	

FM, g (5–30 weeks)	1.647 + (0.053 × age)	1.431 1.862	0.03594 0.07156		0.0001	

**Table 4:** Mean and standard deviation of observed values for repeated DEXA measurements in female C57BL/6J mice from 5 to 9 weeks (group 1, no. 10), from 5 to 21 weeks (group 2, no. 5), and from 13 to 30 (group 3, no. 5) weeks of age groups.

Age (weeks)		5	9	13	17	21	25	30
BMD g/cm^2^	group 1	0.0404 ± 0.0042	0.0452 ± 0.0027					
	group 2	0.0355 ± 0.0007	0.0471 ± 0.0009	0.0499 ± 0.0015	0.0548 ± 0.0011	0.0525 ± 0.0014		
	group 3			0.0482 ± 0.0015		0.0496 ± 0.0022	0.0528 ± 0.0011	0.0510 ± 0.0048

LM, g	group 1	12.87 ± 0.70	15.70 ± 1.01					
	group 2	12.12 ± 0.66	15.80 ± 0.58	16.94 ± 0.79	17.66 ± 0.61	17.85 ± 0.77		
	group 3			17.32 ± 0.78		19.98 ± 0.23	20.94 ± 0.33	21.70 ± 1.39

FM, g	group 1	2.27 ± 0.24	2.08 ± 0.21					
	group 2	1.90 ± 0.07	2.28 ± 0.16	2.64 ± 0.09	3.62 ± 0.66	3.96 ± 0.33		
	group 3			1.84 ± 0.23		2.84 ± 0.63	3.90 ± 0.90	4.20 ± 0.71
